# Cervical solitary fibrous tumor: case report and literature review

**DOI:** 10.1259/bjrcr.20210058

**Published:** 2022-03-09

**Authors:** Camilla Akemi Felizardo Yamada, Eduardo de Oliveira Narvaez, Vitor Nagai Yamaki, Robert Zawadzki Pfann, Iuri Santana Neville, Lázaro Luís Faria do Amaral

**Affiliations:** 1 Departament of Oncology, Hospital BP, São Paulo, Brazil; 2 Latin American Cooperative Oncology Group - LACOG, Sao Paulo, Brazil; 3 Departament of Neuroradiology, Hospital BP, São Paulo, Brazil; 4 Department of Neurosurgery, Universidade de São Paulo, São Paulo, Brazil; 5 Instituto do Cancer do Estado de São Paulo – Hospital das Clínicas da Faculdade de Medicina da Universidade de São Paulo, São Paulo, Brazil

## Abstract

Solitary fibrous tumors (SFTs) are rare neoplasms in the spinal canal. There are few studies addressing SFT/hemangiopericytomas with no distinctive clinical characteristics, no conclusive radiological findings or even a well-defined best treatment strategy. We described a rare case of cervical SFT/hemangiopericytomas in a young patient with spinal cord compression. There are many differential diagnoses for spinal dural-based masses of which meningiomas are the most common. Surgeons and oncologists should be aware of differentials of dural-based masses in the spinal cord for surgical decision making and to guide treatment.

## Introduction

Solitary fibrous tumors/hemangiopericytomas (SFT/HPC) are rare tumors of the central nervous system (CNS) usually presenting as intracranial extraaxial tumors with mesenchymal origin.^1,2^ The spinal SFT/HPC are unusual and might present as extramedullary dural-based tumors.^3^

There are few cases series addressing the spinal SFT/HPC. There is no distinctive clinical characteristics of patients, a wide range in the age of onset, equally distributed between male and female patients, and unspecific neurologic manifestations; such as: local pain, radiculopathy or sensorimotor disturbance.^4^ The radiological characteristics of the SFT/HPC are not conclusive as well. They usually appear as well-circumscribed soft-tissue masses, with iso- to hypointense T1 signal, heterogeneous hyperintense on *T*_2_WI, and solid contrast enhancement.^5,6^

Therefore, the definitive diagnosis is usually confirmed after histological analysis with uniform spindle cells arranged in interlacing fascicles and dense reticulin fibers.^7^ Diffuse positivity for Vimentin and CD34 stain and genetic expression of STAT6 confirms the diagnosis according to 2016 WHO classification of tumors of the central nervous system.^8^

Reports addressing this rare presentation of the SPF/HPC are necessary for gathering knowledge regarding this tumor in a rare site of occurrence. We report a cervical SFT/HPC case with a subacute presentation of spinal cord compression in a young patient. Additionally, a brief literature review is presented for discussion on clinical aspects, treatment, histology, and genetic appearances of spinal SFT/HPC.

### Case report and literature review

**Figure 1.**
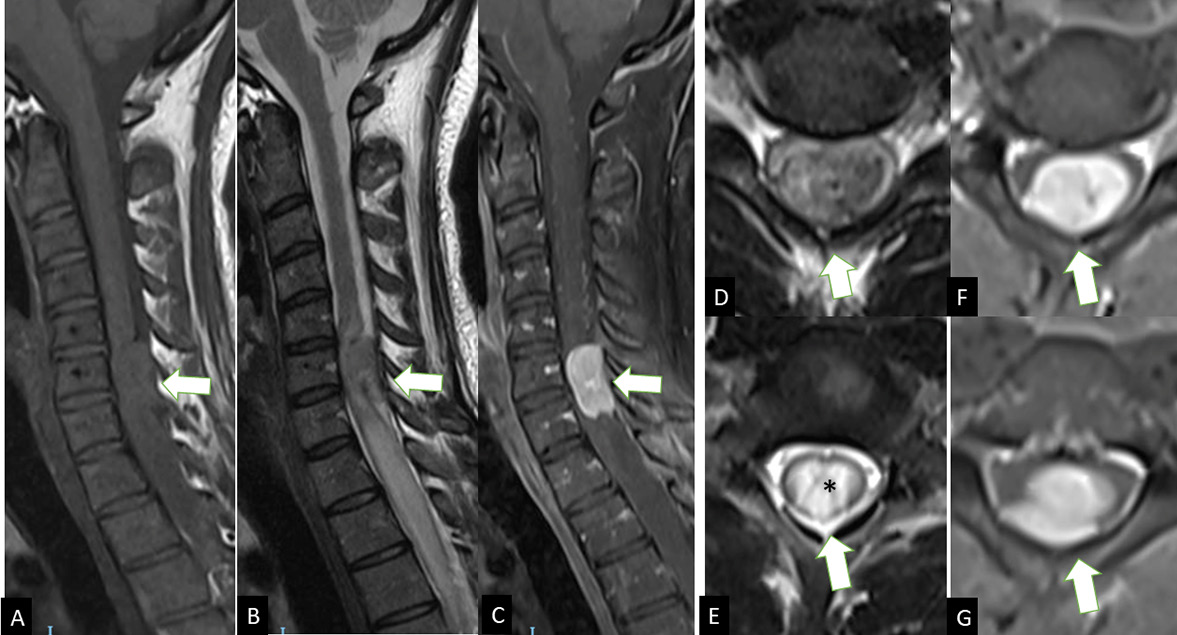
Conventional sagittal MRI images show an intradural and extramedullary lesion (white arrows) with an isointense signal on *T*_1_WI (**A**) and low signal on *T*_2_WI (**B, D**) with nodular enhancement and dural base post-godilinium (**C, F, G**) located in the dorsal and median line of the cervical spinal canal, at the C6–C7 vertebral level. Note the dorsal spinal cord compression and severe canal narrowing, accompanied by pial engorgement and myeloedema (asterisk), extended to thoracic levels (Figures B and E).

**Figure 2.**
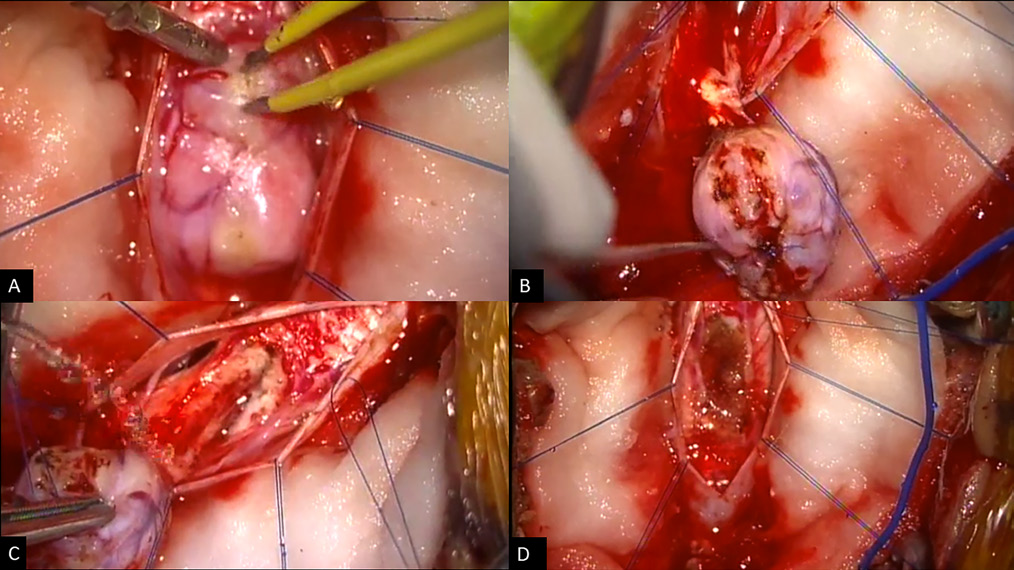
Intraoperative microscopic view showing complete resection of the lesion - SFT/HPC. HPC, hemangiopericytoma;SFT, Solitary fibrous tumor

A 32-year-old female patient presented with subacute onset of reduced strength in four limbs and gait disturbance. On neurological examination, there was a symmetrical Grade IV tetraparesis with hyperactive deep reflexes, positive Hoffman sign, and posterior cord syndrome with loss of proprioception and vibration in inferior limbs. A spinal cord MRI was performed for investigation that revealed a nodular lesion located along the dorsal and median line in the cervical canal, at the C6–C7 vertebral levels. The lesion presented with an isointense signal on *T*_1_WI and hypointensity on *T*_2_WI and short-tau inversion recovery images in addition to an avid contrast enhancement after gadolinium injection. There was an important spinal cord compression with pial engorgement and myeloedema extending towards the thoracic segments of the spinal cord (Figures 1 and 2).

**Figure 3.**
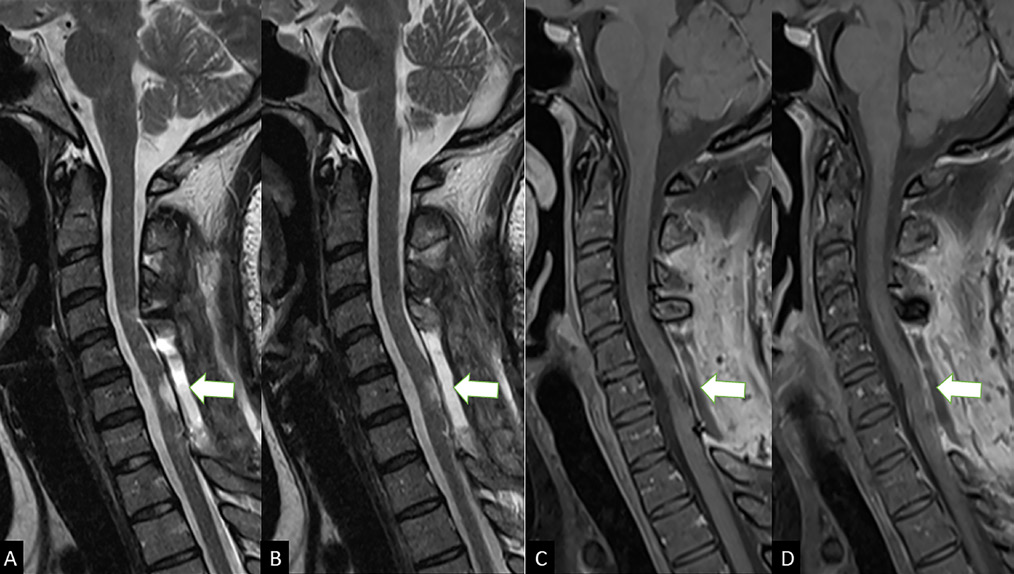
Conventional sagittal MRI images on *T*_2_WI (**A, B**) and *T*_1_WI fat suppression post-gadolinium (**C, D**) post-operative with broad laminectomies in C5–C7 and small surgical cavity in the posterior aspect of the lower cervical spinal cord (C6–C7) (white arrows).

The patient underwent urgent decompression and tumor resection through C5–C7 laminectomy with neurophysiological monitoring. After dural opening, a fibrous intradural extramedullary tumor was identified adherent to the posterior pial surface of the cervical spinal cord. Before tumor resection, a transient reduction in the inferior limbs’ motor potentials was observed, which has completely reversed after tumor removal. The tumor had hard consistency with prominent vascular supply and was totally removed *en bloc* (Figure 3).

**Figure 4.**
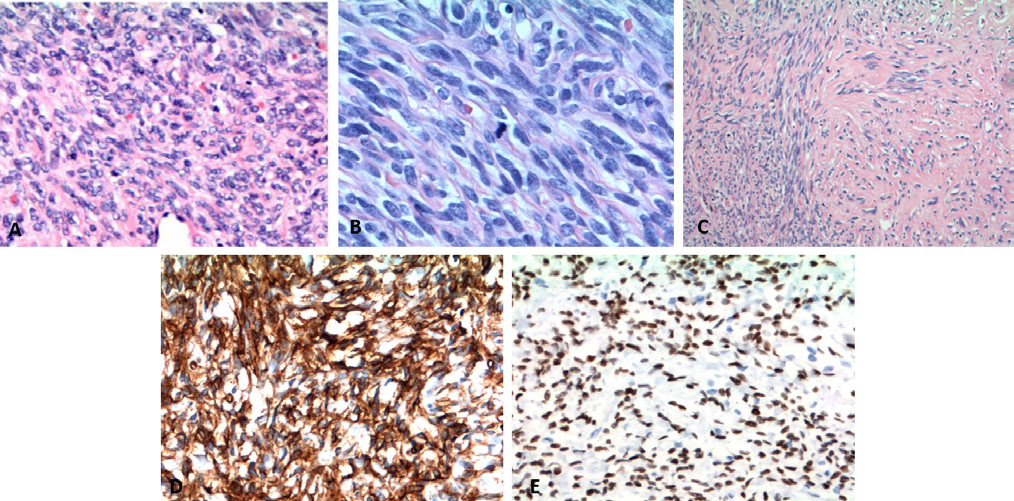
Histologic findings. (**A**) The hematoxylin-eosin stain was demonstrating hypercellular areas and prominent interspersed vessels. (**B, C**) Immunohistochemically staining showing increased mitotic figures and areas of collagen deposition. (**D**) Positive CD34 staining. (**E**) Diffuse nuclear positivity for STAT6 staining.

In the first post-operative, the patient improved tetraparesis and presented partial improvement of gait instability. The pathological analysis revealed a SFT/HPC (WHO Grade III). It was described as hypercellular areas composed by medium-sized cells, less evident nucleoli and scarce cytoplasm whose immunochemistry showed proliferation index (Ki-67) 10% and STAT6 positivity expression (Figure 4). The patient received conventional adjuvant radiotherapy to a dose of 45 Gy in 25 fractions without toxicities. There is no evidence of recurrence at 1-year follow-up.

**Figure 5.**
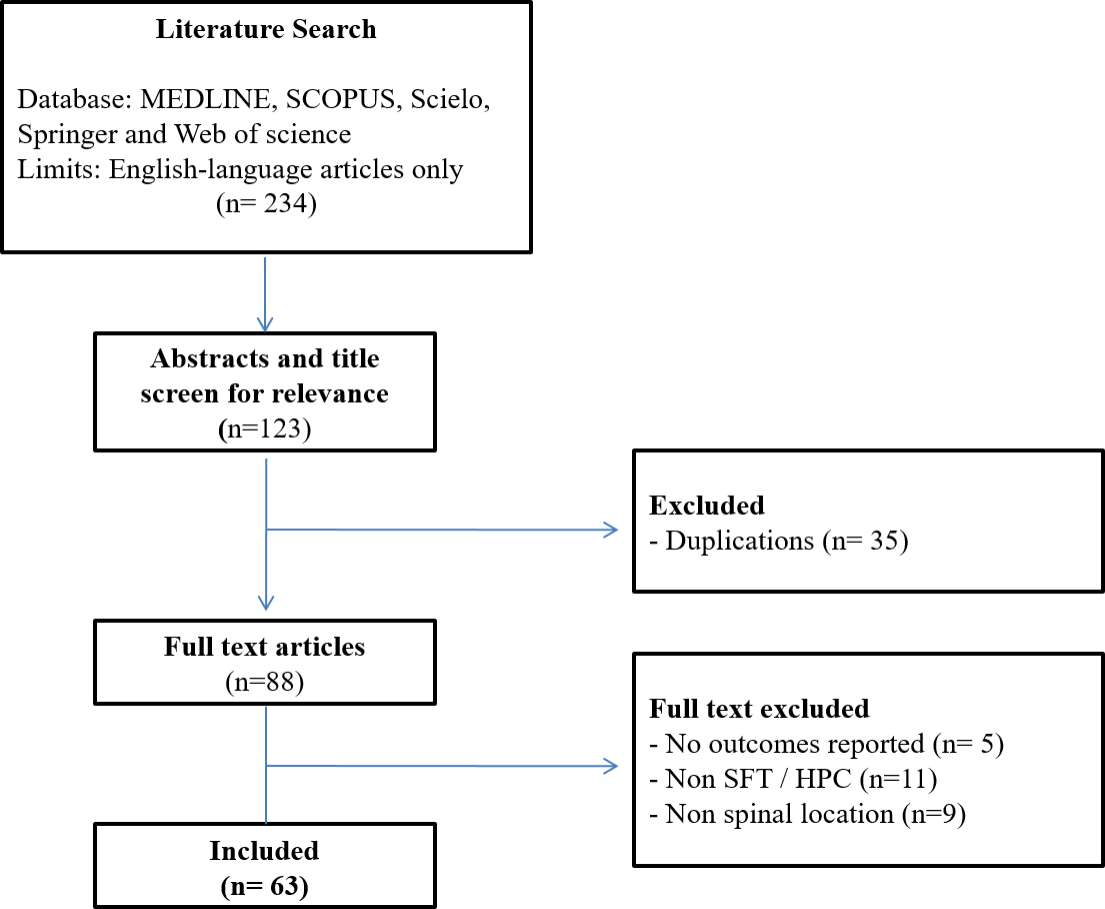
Flow diagram of search strategy and study selection.

We performed a Pubmed search through June/2020. The search terms were: solitary fibrous tumor OR hemangiopericytoma AND spinal. We included 63 publications from 1996 to 2020. There were 3 case series (*n* ≥ 5 patients) and 60 case reports with individual information regarding 108 patients (Figure 5). Table 1 summarizes the main findings from individual data of patients collected. Supplementary Table 1 shows individual data from the studies.

Supplementary Table 1.

**Table 1.** Summarized data of spinal SFT/HPC from literature review

		(%)
**Age (median**)	47.5 years (range 10–86)	
**Male (%**)	52.9%	
**Spinal level**	Cervical Thoracic Lumbar	32.4% 52.9% 13.9%
**Longitudinal extension** (**spinal cord levels**)	one level two levels ≥3 levels	28.3% 52.5% 19.2%
**Location in spinal cord compartment**	Intradural Extramedullary Intramedullary Extradural	69% 23% 8%
**Clinical presentation**	Progressive onset	96.4%
	Pain Sensorial changes Motor deficits Sphincter dysfunction	50% 43.3% 50.5% 5%
**Treatment**	Surgery Gross total resection	100% 73.5%
	Adjuvant treatment RT CT	13.8% 13.8% 5%
**Follow-up** (**months / median**)	29 (range 3–288 months)	
**Recurrence rate (%**) **Recurrence after GTR (%**) **Recurrence after STR (%**)	33.3% 19% 65.3%	

GTR, gross total resection; HPC, hemangiopericytoma; SFT, solitary fibrous tumor; STR, subtotal resection.

## Discussion

SFT/HPC have been recently recognized as a unique entity in the 2016 WHO classification of tumors of the central nervous system with a genetic biomarker corresponding to the fusion of the NAB2 and STAT6 genes at the 12q13 locus.^8^ This tumor is more often seen as differentiation of soft tissue in the pleura with a mesothelial or mesenchymal origin. Its occurrence in the CNS remains controversial; the dural-based SFT/HPC is hypothesized to originate from mesenchymal differentiation of the meninges.^1,3^

SFT/HPC in the spinal cord is an extremely rare site of occurrence.^4,10^ Our review registered more than 100 cases published since the first description in 1996.^1^ The spinal SFT/HPC has no characteristic diagnostic feature in the clinical setting; therefore, it should be included as a differential diagnosis of usual spinal tumors - meningiomas and schwannomas.

Our literature review included 108 patients with spinal SFT/HPC. There was an equal distribution between gender and a wide variety in the age of onset. The vast majority of patients presented with progressive worsening of symptoms with higher prevalence of motor deficits and pain. 80% of tumors were favorable for safe gross total resection, mostly located within two vertebral levels and extramedullary disposition.

The radiological findings on MRI are not conclusive for SFT/HPC.^2^ Meningiomas and schwannomas are the main differentials, but hemangioblastomas, metastatic lesions, primary osseous tumors should also be considered.^11^ SFT/HPC presents as a dural-based mass with an isointense signal on T1 sequence and avid contrast enhancement in most cases.^12^ However, there are some particular findings in SFT MR images that include the involvement of adjacent bone with osseous erosion instead of hyperostosis, hypervascular nature of the lesion on MR angiography with prominent flow-voids on *T*_2_ weighted images (*T*_2_WI), and absence of intratumoral calcification. Additionally, some cases have marked heterogeneous areas of hypointensity areas on *T*_2_WI representing fibrous tissue with different components inside the tumor.^11^

All cases included in our review underwent surgical treatment. Gross total resection was achieved in more than 70% of patients, and adjuvant therapy was required in 13.8%, mostly radiation therapy. At 29 months follow-up, 65% of patients with residual tumors presented local recurrence. The GTR has been considered the only outcome predictor for disease free-survival. Although there is weak evidence based on small case series and case reports, there is no clear benefit from adjuvant therapy. Wang et al^4^ suggested a benefit of local radiation after surgical resection in recurrence-free survival and overall survival; however, only 6 out of 18 patients underwent RT, 5 of them had a gross total resection.

The risk of recurrence should be considered according to the tumor’s biological behavior^2,13^; however, the natural history of these tumors remains unknown.^13^ Higher tumor grade or Ki67 expression might not be related to a higher risk of recurrence.^2,13^ Approximately, 10–15% of extra pleural SFT have an aggressive behavior with a high recurrence rate and distant metastasis.^14^ Adjuvant treatment should be encouraged in the treatment of recurrent tumors since there is no consensus on managing of such aggressive tumor.^3,4^

Our study has limitations. There were some missing data from papers included in our review which resulted in some missing information. The heterogeneity of data collected is limited to perform an analytical analysis for more accurate conclusions. However, we presented an interesting case of cervical SFT/HPC case with subacute onset of spinal cord compression, effective treatment with surgical resection followed by radiation therapy and no recurrence at 1-year follow-up. Our review shows a global view of the current status of spinal SFT/HPC’s current status with relevant information regarding its clinical, radiological presentation and treatment.

## Learning points

1. Spinal SFT/HPC are extremely unusual tumors. It should be considered as differential diagnosis of more common spinal tumors such as meningiomas.
2. Clinical and radiological findings of SFT/HPC are inconclusive; the diagnosis confirmation requires detailed histologic and immunohistochemistry study. The current biomarker is the expression of the *STAT6* gene
3. Surgical treatment with safe gross total resection is the state-of-the-art treatment; radiotherapy seems to have benefit and should be considered for recurrent tumor. The biological behavior of the tumor remains undetermined with no correlation with histological findings.
