# Supplementary material for: Cervical solitary fibrous tumor: case report and literature review
Source: BJR Case Rep. 2022 Mar 9;7(6):20210058. doi: 10.1259/bjrcr.20210058 (PMC8906161; doi:10.1259/bjrcr.20210058)
Supplement: Supplementary Table 1. [file bjrcr.20210058.suppl-01.docx]

Supplementary table – 63 studies included

| **Author, year** | **n** | **Median age (range)** | **Female (%)** | **Cervical (n)**  **Thoracic (n)**  **Lumbar (n)** | **Vertebral compartment** | **Onset symptoms** | **GTR (%)** | **Adjuvant treatment** | **Recurrence (%)** | **Follow-up (months)** |
| --- | --- | --- | --- | --- | --- | --- | --- | --- | --- | --- |
| Fernandez et al, 1979 | 1 | 51 | Male | Thoracic | Intradural extramedullary | - | GTR | - | - | 7 |
| Carneiro et al, 1996 | 2 | 52 (50-54) | Female 100% | Lumbar | Intramedullary | Motor 50%  Sensorial 100%  Pain 50% | GTR 50%  STR 50% | None | 50% | 78 (72-84) |
| Malek et al, 1997 | 1 | 33 | Male | Thoracic | Intradural extramedullary | Motor  Sensorial  Pain | GTR | - | - | - |
| Brunori et at, 1999 | 2 | 32 (18-46) | Female 50% | Cervical 50%  Thoracolumbar 50% | Intradural extramedullary | Motor 50%  Sensorial 100%  Sphincter 50% | GTR | None | None | 8 (4-12) |
| Kanahara et al, 1999 | 1 | 62 | Male | Cervical | Intradural extramedullary | Sensorial | - | - | - | - |
| Kataoka et al, 1999 | 1 | 46 | Male | Cervical | Intradural extramedullary | Pain  Sensorial | - | - | - | - |
| Hasegawa et al, 1999 | 1 | 39 | Make | - | Extradural | - | - | - | Yes | 108 |
| Vorster et al, 2000 | 1 | 51 | Male | Thoracic | Intradural extramedullary | Motor  Sensorial | GTR | None | None | 7 |
| Donnellan et al, 2000 | 1 | 39 | Male | Lumbar | Extradural | Pain | GTR | - | - | - |
| Mordani et al, 2000 | 1 | 33 | Male | Cervical | Intramedullary | Sensorial | GTR | None | None | 18 |
| Kurtaya et al, 2001 | 1 | 70 | Female | Thoracic | Intradural extramedullary | Motor | GTR | None | None | 12 |
| Tihan et al, 2003 | 4 | - | - | - | Intramedullary | - | - | - | - | - |
| Obara et al, 2003 | 1 | 49 | Female | Cervical | - | Motor | GTR | None | None | 12 |
| Endo et al, 2003 | 1 | 63 | Female | Cervical | Intradural extramedullary | Sensorial  Pain | - | - | None | - |
| Caroli et al, 2004 | 1 | 54 | Male | Cervical  Thoracic | Intradural extramedullary | Motor  Sensorial  Pain | GTR | None | None | 15 |
| Pizzolito et al, 2004 | 2 | 41.5 (36-47) | Male 100% | Cervical 50%  Thoracic 50% | Intradural extramedullary | Sensorial | GTR | - | - | - |
| Piana et al, 2004 | 1 | 67 | Male | Lumbar | Extradural | Motor  Pain | GTR | - | - | - |
| Bohinski et al, 2004 | 1 | 49 | Female | Cervical | Intradural extramedullary | Pain | GTR | None | None | 10 |
| Kawamura et al, 2004 | 1 | 64 | Male | Thoracic | Intradural extramedullary | Motor | STR | None | None | 6 |
| Pakasa et al, 2005 | 1 | 27 | Male | Thoracic | Intradural extramedullary | Pain | STR | None | Yes | 168 |
| Jallo et al, 2005 | 4 | 38.5 (17-59) | Male 75%  Female 25% | Cervical 25%  Thoracic 75% | Intradural extramedullary 50%  Intramedullary 50% | Motor 50%  Sensorial 75% | GTR | None | None | 44.5 (19-60) |
| Ogawa et al, 2005 | 1 | 63 | Female | Thoracic | - | Sensorial | GTR | None | None | 10 |
| Ogungbo et al, 2005 | 1 | 53 | Male | Thoracic | Intradural extramedullary | Motor  Sensorial  Pain | GTR | - | - | - |
| Metellus et al, 2007 | 6 | 55 (44-72) | Male 50%  Female 50% | Cervical 16.6%  Thoracic 83.3%  Lumbar 16.6% | - | Motor 33.3%  Pain 66.6% | GTR 83.3%  STR 16.6% | None | 33.3% | 40.6 (9-102) |
| Shin et al, 2008 | 1 | 40 | Female | Cervical | Extradural | Motor  Pain | GTR | None | None | 8 |
| Muñoz et al, 2008 | 1 | 35 | Male | Sacral | Intradural extramedullary | Sensorial | GTR | None | Yes | 102 |
| Hashimoto et al, 2008 | 1 | 71 | Female | Cervical | - | Pain | STR | None | Yes | 48 |
| Arantes et al, 2009 | 1 | 22 | Male | Thoracic | Intradural extramedullary | Motor  Sensorial  Pain | GTR | None | None | 18 |
| Kakiharu et al, 2009 | 1 | 75 | Female | Thoracic | - | Motor | - | None | None | 36 |
| Ishii et al, 2009 | 1 | 63 | Female | Cervical | Intramedullary | Pain | GTR | None | None | 14 |
| Ciappetta et al, 2010 | 1 | 75 | Female | Thoracic | Intradural extramedullary | Motor  Sensorial | GTR | None | None | 12 |
| Aftab et al, 2010 | 1 | 38 | Male | Lumbossacral | Extradural | Pain | STR | None | None | 6 |
| Bisceglia et at, 2011 | 1 | 47 | Male | Thoracic | Intradural extramedullary | Motor | GTR | None | None | 138 |
| Vassal et al, 2011 | 1 | 52 | Female | Thoracic | Intradural extramedullary | Pain | GTR | None | None | 48 |
| Mariniello et al, 2012 | 2 | 71 (67-75) | Female 50% | Cervical 50%  Thoracic 50% | Intradural extramedullary | Motor  Sensorial 50% | GTR | None | None | 12 (12) |
| Wu et al, 2012 | 1 | 50 | Female | Lumbar | Intramedullary | Pain | GTR | - | - | - |
| Chen et al, 2012 | 2 | 53 (43-63) | Male | Thoracic 50%  Lumbar 50% | - | Motor 100% | GTR | - | 50% | 104.5 (33-176) |
| Brigui et al, 2013 | 1 | 56 | Male | Thoracic | Intradural extramedullary | Pain | GTR | None | None | 29 |
| Robert et al, 2013 | 1 | 49 | Female | Thoracic | Intramedullary | Motor  Sensorial | STR | None | None | 6 |
| Drazin et tal, 2013 | 1 | 56 | Male | Cervical | Intradural extramedullary | Pain | GTR | None | Yes | 60 |
| Nakashima et al, 2013 | 1 | 51 | Female | Cervical | Extradural | Pain | STR | None | Yes | 108 |
| OpdeBeek et al, 2013 | 1 | 49 | Female | Thoracic | Intradural extramedullary | Motor  Sensorial | STR | RT | None | 6 |
| Hwang et al, 2014 | 1 | 48 | Male | Cervicothoracic | Intradural extramedullary | Sensorial | STR | None | None | 6 |
| Robert et al, 2014 | 1 | 49 | Female | Thoracic | Intradural extramedullary | Motor  Sensorial | STR | None | None | 6 |
| Yuan et al, 2014 | 1 | 48 | Male | Thoracic | Intradural extramedullary | Sensorial | GTR | None | - | - |
| Kobayashi et al, 2014 | 1 | 40 | Male | Cervical | Intradural extramedullary | Motor  Sensorial | STR | None | Yes | 288 |
| Tomomatsu et al, 2014 | 1 | 68 | Female | Thoracic | Intradural extramedullary | Sensorial  Pain | GTR | None | None | 36 |
| Nagano et al, 2014 | 1 | 57 | Female | Lumbar | Extradural | Pain | GTR | None | Yes | 3 |
| Sade et al, 2015 | 1 | 43 | Male | Thoracic | Intradural extramedullary | Motor | - | - | - | - |
| Das et al, 2015 | 5 | 37 (12-50) | Female 40% | Cervical 40%  Thoracic 60% | Intradural extramedullary | Motor 100%  Sensorial 40%  Sphincter 20% | 60% | RT 80%  ChT 60% | 40% | 24 (6-120) |
| Bruder et al, 2015 | 1 | 83 | Female | Thoracic | Intradural extramedullary | Motor  Sensorial | GTR | None | None | 8 |
| Walker et al, 2015 | 1 | 47 | Female | Lumbar | Intramedullary | Pain | GTR | None | None | 12 |
| Wang et al, 2016 | 1 | 31 | Male | Thoracic | Intramedullary | Motor  Sensorial | GTR | - | - | - |
| Albert et al, 2017 | 1 | 10 | Male | Cervical | Intradural extramedullary | Motor | GTR | None | None | 12 |
| Biswas et al, 2017 | 1 | 35 | Female | Thoracic | Intradural extramedullary | Motor  Pain | STR | None | Yes | - |
| Yu et al, 2017 | 3 | 45.6 (31-64) | Male 66.6%  Female 33.3% | Thoracic 66.6%  Lumbar 66.6%  Sacral 33.3% | - | Motor 33.3%  Pain 66.6%  Sphincter 33.3% | GTR | None | None | 49 (36-62) |
| Jiang et al, 2017 | 4 | 38 (26-37) | Male 75%  Female 25% | Cervical 25%  Thoracic 25%  Lumbar 50%  Sacral 25% | - | Motor 25%  Sensorial 25%  Pain 100%  Sphincter 25% | GTR 75%  STR 25% | - | 50% | 32.5 (3-64) |
| Flores-Justa et al, 2018 | 1 | 63 | Female | Thoracic | Intradural extramedullary | Motor | STR | None | None | 12 |
| Wang et al, 2019 | 16 | 42.5 (21-66) | Male 37.5%  Female 62.5% | Cervical 25%  Thoracic 56.25%  Lumbar 18.75% | Intradural extramedullary 62.5%  Intramedullary 37.5% | Motor 25%  Sensorial 12.5%  Pain 93.75%  Sphincter 6.25% | GTR 56.25%  STR 43.75% | RT 31.25%  None 68.75% | 50% | 63.2 (24-96) |
| Yang et al, 2019 | 1 | 35 | Female | Cervicothoracic | Intramedullary | Sensorial | GTR | None | None | 23 |
| Glauser et al, 2020 | 1 | 72 | Male | Cervical | Intradural extramedullary | Motor  Sensorial  Pain | GTR | - | - | - |
| Carlston et al, 2020 | 3 | 74.6 (65-86) | Male 33.3%  Female 66.6% | Cervical 66.6%  Thoracic 33.3% | Intradural extramedullary | Motor 66.6%  Sensorial 66.6%  Pain 33.3% | GTR 66.6%  STR 33.3% | RT 33.3%  None 66.6% | None | 9 (6-12) |
| Dauleac et al, 2020 | 1 | 67 | Male | Thoracic | Intradural extramedullary | Motor  Sensorial | GTR | None | None | 12 |

GTR- gross total resection

STR - subtotal resection
